# The Result Is Clear: Color Trap Preferences of Adult Necrophagous Flies

**DOI:** 10.3390/biology15070519

**Published:** 2026-03-25

**Authors:** Aidan E. Bonn, Karielly L. Castaneda, Clara L. Stump, Edward B. Mondor, Evan C. Lampert

**Affiliations:** 1 Department of Biology, University of North Georgia, Oakwood, GA 30566, USA; 2 Department of Biology, Georgia Southern University, Statesboro, GA 30458, USA; emondor@georgiasouthern.edu; 3 Center for Forensic Sciences, Georgia Southern University, Statesboro, GA 30458, USA

**Keywords:** forensic entomology, preference, Calliphoridae, bottle trap, attraction, spectral reflectance

## Abstract

Color vision plays a critical role in Diptera behavior, particularly in the detection of oviposition sites. Necrophagous Diptera, like the Calliphoridae, are of forensic importance because the larvae are commonly used to estimate minimum postmortem intervals of human remains. To better understand which species are present, flies are routinely sampled in different habitats using baited traps; however, the influence of trap color on capture efficiency remains poorly understood. In this study, baited bottle traps painted clear, blue, red, and yellow were deployed in wooded habitats to collect adult dipterans. Overall captures were dominated by *Lucilia coeruleiviridis* (Diptera: Calliphoridae). Clear traps consistently captured a greater diversity of Calliphoridae, Muscidae, and Sarcophagidae. In contrast, yellow traps captured the fewest individuals overall, while red and blue traps yielded intermediate numbers. Dipteran composition in red and yellow traps, however, differed from those in clear traps. Collectively, these results indicate that clear baited traps are more effective for sampling adult necrophagous Diptera, whereas yellow baited traps may be less suitable. Additional investigation is warranted to characterize the complex interactions between visual and olfactory cues underlying attraction and oviposition site selection in necrophagous dipterans.

## Simple Summary

Blow flies and other flies that utilize animal remains are frequently sampled for both basic and applied research. Sampling techniques vary; for adults, baited traps such as colored sticky paper and bottle traps are often used to assess species presence and abundance. Little is known about how the color of baited bottle traps affects fly sampling. The objective of this study was to compare the species composition, abundance, and sex ratio of flies captured in clear-, blue-, red-, and yellow-painted bottle traps. Clear bottle traps captured more individuals and species than the yellow bottle traps, with blue and red capturing intermediate numbers. This study supports the use of clear bottle traps for sampling adult flies and does not support the use of yellow bottle traps. Based on this study, it is recommended that ecologists and forensic investigators use clear bottle traps when assessing fly species distributions in various habitats.

## 1. Introduction

Color vision, the ability to detect and differentiate wavelengths of light, is widespread among insects [1,2] and has been shown to influence behaviors such as foraging, mate selection, and oviposition [3]. Color cues can facilitate resource detection and assessment in visually complex environments by allowing insects to locate, recognize, and assess the suitability of resources [4]. In Diptera, visual cues often act in combination with olfactory cues to mediate attraction, landing, and resource use [5].

Adult necrophagous dipterans utilize both floral resources for feeding and animal remains for feeding and oviposition [6]. Odor cues generally operate at longer distances, whereas visual cues appear to become more important near the food source or oviposition substrate [6,7]. Color vision may facilitate exploitation of a range of resources, from colorful nectar-bearing flowers to less conspicuous substrates such as sapromyophilous flowers and decomposing remains [3]. Necrophagous dipterans have also been shown to learn and associate colors with rewards, further indicating the importance of these stimuli [8].

Despite evidence that color can function as an important visual cue for many necrophagous Diptera, studies have produced variable and sometimes conflicting results. Calliphoridae, the most studied family, can discriminate among color wavelengths [3,5,9,10]. Choice experiments with *Lucilia sericata* (Meigen) (common green bottle fly) and *Calliphora vomitoria* L. (blue bottle fly) found preferences for dark colors, including black [6,11,12], which have been hypothesized to occur due to high contrast [7,13]. On the contrary, other studies with the *L. sericata* and *L. cuprina* (Wiedemann) (Australian sheep blow fly) report preferences for bright colors such as white and yellow [9,14,15], possibly reflecting associations between nectar and pollen rewards advertised by floral displays [11]. Red colors were reported as less attractive to *L. sericata* [15] and calliphorid communities [16], despite the reddish appearance of hemoglobin- and myoglobin-rich animal remains to human observers, potentially due to a limited sensitivity to longer wavelengths in many insects [17]. Field studies examining calliphorid communities remain inconclusive, reporting either no color preference [18] or species-specific variation for different trap colors [19,20], including clear traps over other colors [21]. Such variability may reflect intrinsic differences among species or populations, methodological factors, especially bait type, or other environmental conditions not readily apparent to researchers.

Accurate sampling of necrophagous dipterans is important for both basic and applied research, including medicolegal forensic entomology [22]. These insects provide important ecosystem services such as nutrient cycling [23] and are frequently sampled to better understand the species involved in decomposition ecology. Necrophagous dipterans, particularly Calliphoridae, typically arrive at animal remains rapidly to feed and oviposit [24]. Although larval development is key to estimating minimum postmortem intervals [25,26], sampling adults, often more easily identifiable, assists in characterizing the local community [27]. Previous field studies examining the influence of color on sampling outcomes have used a range of baited trap types, including colored plastic bottle traps [21], painted bottle or pipe traps [18], and colored sticky traps [18].

While bottle traps are commonly used to sample necrophagous dipterans [28,29,30], there exists a knowledge gap in the extent to which trap color influences sampling outcomes. The objective of this study was to assess whether species composition, abundance of common taxa, and sex ratio differ among bottle traps painted clear or with the three primary colors. We hypothesized that species would differ in their preferences for trap color. Specifically, we hypothesized that: (1) blue traps would capture higher numbers of individuals, as dark colors have previously been reported to attract calliphorids seeking oviposition substrates, and (2) the sex ratio of calliphorids would vary among trap colors, with yellow traps having a more female-biased sample, reflecting the use of floral rewards such as pollen for egg maturation [11]. This study aims to improve our understanding of how color stimuli influence necrophagous dipteran sampling, with implications for both ecological research and forensic applications.

## 2. Materials and Methods

### 2.1. Field Sampling

Data collection was conducted from 26–31 August 2024, at two forested sites in Lumpkin and Hall counties in north Georgia. At the Hall County site (34°14′36″ N, 83°51′41″ W), dominant overstory species included *Quercus nigra* (water oak), *Acer rubrum* (red maple), *Liriodendron tulipifera* (tulip poplar), and *Liquidambar styraciflua* (sweetgum). The understory and groundcover were primarily *Muscadinia rotundifolia* (muscadine grape) and *Vaccinium pallidum* (highbush blueberry). Average daily high and low temperatures, recorded at the Oakwood Weather Station located > 1 km from the site, were 34.20 °C ± 0.52 and 18.78 °C ± 1.00, respectively. At the Lumpkin County site (34°31′07″ N, 84°03′55″ W), dominant overstory species included *Alnus serrulata* (smooth alder), *Acer rubrum* (red maple), and *Pinus taeda* (loblolly pine). The understory consisted of *Arundinaria gigantea* (river cane), *Ligustrum sinense* (Chinese privet), *Dryopteris cristata* (crested wood fern), *Dryopteris cristata* (cinnamon fern), and *Microstegium vimineum* (Japanese stilt grass). Average daily high and low temperatures, recorded at the Three Sisters Weather Station approximately 35 km from the site, were 32.98 °C ± 0.53 and 17.96 °C ± 0.82, respectively.

Dipterans were sampled using bottle traps constructed from 2 L and 591 mL clear polyethylene terephthalate (PET) soda bottles connected with plastic cyclone tubes; see Mondor et al. [28,29]. Bottles were painted with Rust-oleum^®^ American Accents satin spray paints in clear, ink blue, sweet mango (yellow), or apple red and the cyclone tubes were wrapped with white duct tape. The 2 L bottles were baited with raw chicken drumsticks and the 591 mL bottles contained approximately 100 mL of 70% ethanol to kill and preserve captured insects.

Traps were hung in groups of four, one of each color, within 5 m of each other. Each group was replicated five times at both forest sites, resulting in a total of 40 traps. Traps were hung approximately 1 m above the ground, and clusters were spaced approximately 30 m apart to prevent insects from viewing multiple clusters simultaneously.

After collection, the catch was sorted and identified to family, with Calliphoridae further identified to species [31]. The sex of each calliphorid was determined by frons width, as males have a narrower frons than females [32].

### 2.2. Trap Spectral Reflectance Measurements

Spectral reflectance of the painted bottle traps was measured using a Nix Spectro 2 spectrophotometer (Nix Sensor Ltd., Hamilton, ON, Canada). For each paint color, four swatches (approximately 2 × 3 cm) were cut from the sides of the bottles and placed on a matte black cardstock background (“Eclipse Black,” Astrobrights, Wausau Paper Mills LLC, a subsidiary of Neenah Paper, Neenah, WI, USA) to minimize background reflectance and edge effects. Each swatch was scanned individually, with the instrument recording proportional reflectance at 10 nm intervals across the visible spectrum (400–700 nm). An unpainted bottle was also measured for comparison with the clear-painted bottles. For each color, four readings were obtained, and mean reflectance at each wavelength was calculated and plotted to represent the spectral profile of a typical trap.

**Figure 1:**
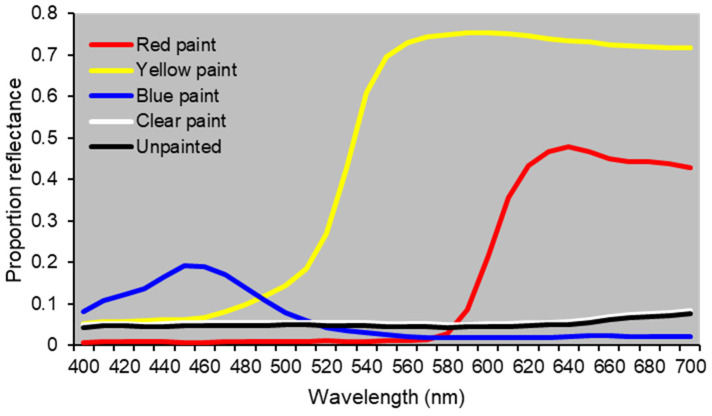
Spectral reflectance of the painted bottle traps.

The spectral reflectance of the painted bottle traps differed among colors (Figure 1). Blue-painted traps exhibited lower overall reflectance compared to red and yellow traps, with a peak at 440–460 nm and minimal reflectance above 560 nm. Red-painted traps showed the highest reflectance at longer wavelengths, above 620 nm. Yellow-painted traps had the highest overall reflectance, exceeding 0.70 at wavelengths above 550 nm. Reflectance did not differ between clear-painted and unpainted bottles.

### 2.3. Data Analysis

The composition of dipteran taxa recovered in the traps was compared among the four trap colors using non-metric multidimensional scaling (nMDS). Abundances of each taxon in each trap were standardized to relative abundance and square-root transformed prior to analysis. Bray–Curtis similarity was used as the resemblance measure. The nMDS scatterplot was visualized in two dimensions to minimize stress, with 25, 50, and 75% similarity overlays. Significant differences among trap colors were identified using analysis of similarity (ANOSIM). For pairwise comparisons that were significant (*p* < 0.05), similarity percent (SIMPER) was conducted to determine which taxa contributed most to composition differences between trap colors. All multivariate analyses were performed using PRIMER7 (PRIMER-e, Auckland, NZ, New Zealand).

Trap catch data were analyzed using negative binomial generalized linear models (GLMs), with the number of individuals in each trap as the response variable and trap color and trap group (hereafter referred to as “block”) as independent variables. The “block” variable was included to account for non-independence among the four traps hung together. Sampling site was included in initial models as a random factor, but it was not significant in any model (*p* > 0.60 in all models) and removed to limit overfitting the models. When models were significant, pairwise comparisons were used to identify which trap color(s) differed in the numbers of dipterans collected. Analyses were performed separately for each family and each calliphorid species using the GLM procedure in SPSS v.29 and v.31 (IBM, Armonk, NY, USA).

The effects of sex were analyzed using binomial GLMs, with sex (male or female) as the response variables and trap color as the independent variable. Analyses were performed separately for each calliphorid species using the GLM procedure in SPSS v.29 and v.31.

## 3. Results

Diptera were the most commonly collected insects, comprising 73% (977/1345) of all captures. Nearly 96% of dipterans belonged to the families Calliphoridae, Muscidae, and Sarcophagidae. Three calliphorid species were identified: *Lucilia coeruleiviridis* Macquart (green bottle fly), *Cochliomyia macellaria* F. (secondary screwworm), and *Phormia regina* Meigen (black blow fly). A total of 687 *L. coeruleiviridis* were collected, representing 70% of dipterans and 51% of the total catch. Four other calliphorids were collected that could not be identified to species; these individuals were, however, still included in the analysis of calliphorid abundance. Four other insect families were collected: hister beetles (Histeridae: Coleoptera), rove beetles including burying beetles (Staphylinidae: Coleoptera), yellow jackets (Vespidae: Hymenoptera), and ants (Formicidae: Hymenoptera).

**Figure 2:**
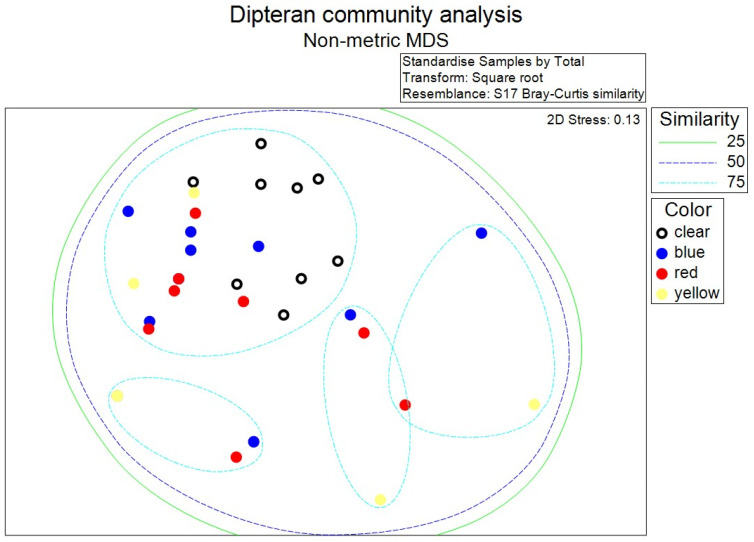
Scatterplot of necrophagous dipteran community composition analyzed using non-metric multidimensional scaling (nMDS). Dipteran samples captured using red and yellow bottle traps were different from those captured using clear traps as shown by their distances in the plot.

Five taxa, *L. coeruleiviridis*, *C. macellaria*, *P. regina,* Muscidae, and Sarcophagidae, were included in the composition analysis. nMDS showed all traps fell within 50% similarity, while four groups with 75% similarity were resolved. Communities from clear traps clustered most closely with communities from blue and red traps and least closely to communities from yellow traps (Figure 2). Dipteran composition differed, but differed weakly, among trap colors according to ANOSIM (R = 0.10, *p* = 0.026). Pairwise comparisons indicated significant differences between clear traps and both red (R = 0.15, *p* = 0.039) and yellow (R = 0.36, *p* = 0.003) traps. These differences were largely driven by the relative abundances of non-dominant species. Almost 80% of the difference between clear and red traps (77.27%) and clear and yellow traps (77.24%) was due to *Phormia regina* (more abundant in red and yellow traps) and Muscidae and Sarcophagidae (more abundant in clear traps). These minority taxa disproportionately influenced the SIMPER analysis because their absences from some trap colors changed species richness values, which alters Bray–Curtis dissimilarities more than abundance of a given species.

**Figure 3:**
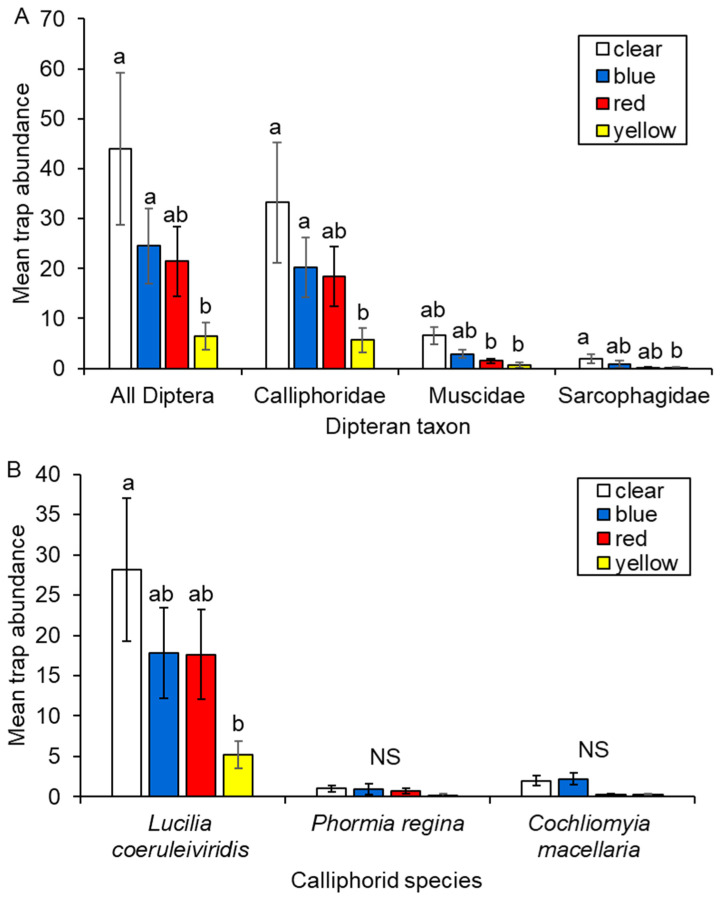
Mean (±SEM) abundances of dipteran families (**A**) and calliphorid species (**B**) captured in baited bottle traps painted four colors. Different lowercase letters indicate significant pairwise differences (*p* < 0.05) based on generalized linear models. NS: Not Significant.

The number of dipterans collected differed among trap colors (Table 1). Fewer individuals were captured in yellow traps compared to blue (*p* = 0.036) and clear traps (*p* = 0.019), with red traps yielding intermediate numbers (Figure 3A). Patterns of color preference differed slightly among the three families.

For Calliphoridae, fewer individuals were captured in yellow traps compared to blue (*p* = 0.047) or clear traps (*p* = 0.026; Figure 3A). For Muscidae, yellow traps captured fewer individuals than blue (*p* = 0.044) and clear traps (*p* = 0.014), while red traps also captured fewer individuals than clear traps (*p* = 0.039; Figure 3A). For Sarcophagidae, pairwise comparisons could not be made due to this family’s absence from several traps. Parameter estimates indicated that fewer individuals were captured in red (*p* = 0.008) and yellow (*p* = 0.008) traps compared to clear traps (Figure 3A).

Within Calliphoridae, the number of *L. coeruleiviridis* differed among trap colors, with yellow traps capturing fewer individuals compared to clear traps (*p* = 0.035; Figure 3B). Numbers of *C. macellaria* and *P. regina* did not differ significantly among trap colors, likely due to very low abundance (Figure 3B).

**Figure 4:**
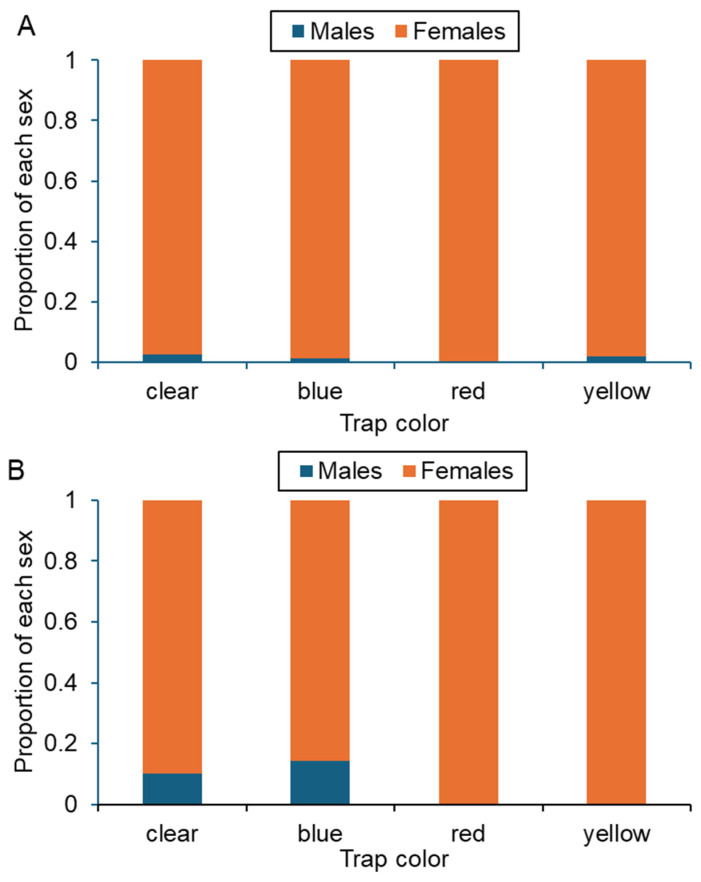
Sex ratios of adult (**A**) *Lucilia coeruleiviridis* and (**B**) *Cochliomyia macellaria* captured in bottle traps painted four colors.

Female calliphorids were more frequently collected than males, comprising over 98% (720/732) of the total catch (Figure 4). All 26 *P. regina* individuals were females, so sex could not be compared among trap colors. Trap color did not significantly affect the chances of capturing either sex for *L. coeruleiviridis* (Wald χ^2^_3_ = 1.012, *p* = 0.798; Figure 4A) or *C. macellaria* (Wald χ^2^_3_ = 0.072, *p* = 0.995; Figure 4B).

## 4. Discussion

Adult necrophagous flies are routinely sampled in different areas to better understand which larval species may be encountered in forensic investigations [27]. The effectiveness of sampling can be largely influenced by trap design and methodology. In this study, captures were significantly affected by bottle trap transparency and color, with different taxa present in traps painted clear and yellow. However, given the low ANOSIM R-value (<0.25), the observed differences among samples may be statistically significant, but may not be biologically significant. Unexpectedly, more necrophagous dipterans were collected in clear-painted compared to yellow traps, a trend observed across *Lucilia coeruleiviridis,* muscids, and sarcophagids. No significant differences were observed between clear-painted and blue traps for any group, falsifying the hypothesis that blue traps would capture the most dipterans. Although trap color did not significantly influence the numbers of *C. macellaria* or *P. regina* sampled, low sample sizes likely limited the ability to detect differences. Clear-painted traps were unique in allowing a visual cue of the bait itself, which may explain their higher attractiveness; previous work similarly found clear traps more attractive than green-colored PET bottle traps [21].

*Lucilia coeruleiviridis* was the dominant species collected, comprising the majority of the catch. This is only the second study, after Oke [21], to investigate color preference and confirm attraction to clear or colorless traps in this species. These results contrast with studies finding yellow is attractive to congeners *L. sericata* [11,15,20] and *L. cuprina* [8]. Differences may reflect intrinsic species- or population-level variation, modifications in trap type (e.g., paper versus bottle), or the specific colors of paint. Ultraviolet (UV) light is highly attractive to calliphorids [11]; the ultraviolet reflectance of the paints in this study are unknown, which could also influence attraction. Based on these results, clear bottle traps that also allow the bait to remain visible are preferable to yellow traps when sampling *L. coeruleiviridis* in areas where this species is common.

Yellow and red are long-wavelength colors and may be less distinguishable and attractive to adult dipterans than shorter-wavelength colors such as blue. Calliphorids have trichromatic vision, with photoreceptors sensitive to blue, green, and UV [3,9,10]. Wavelengths above 550 nm, corresponding to yellow and red, are associated with relatively low photoreceptor sensitivity in Calliphoridae and Muscidae [3]. The yellow-painted traps in this study primarily reflected light above 550 nm, which may not have elicited strong visual responses in some species. Red-painted traps reflected light above 620 nm and attracted more individuals than yellow traps, possibly due to undetected UV reflectance or other factors.

Responses to yellow in previous studies have been inconsistent, with some dipterans showing innate attraction and others showing little or no preference. While yellow can be a signal to dipterans seeking floral resources [6], lack of attraction has been reported in species such as *Musca domestica* L. (the house fly, Muscidae) [33], *Lipoptena cervi* L. (deer keds, Hippoboscidae) [34], *Glossina fuscipes* (tsetse flies, Glossinidae) [35], and several Tabanidae (deer flies) and Simuliidae (black flies) [36,37]. Lack of yellow attraction may occur when this cue is not ecologically relevant to oviposition or foraging.

Female calliphorids were disproportionately caught in this study (>98%), likely reflecting the traps’ primary attraction to adults seeking oviposition substrates. While both sexes seek out floral resources and can respond to color cues [11], gravid females are more likely to visit animal remains for egg maturation and oviposition [26]. Previous work has found sex differences in response to visual contrast cues in *Lucilia* spp. and *Calliphora* spp. [38], but this study found no evidence that color cues differentially attracted males and females, falsifying the hypothesis that yellow bottle traps would preferentially attract females compared to other colors. Inferences about male attraction are limited, however, due to the low number of males sampled in this study. Additional behavioral assays are required to determine whether males exhibit color preferences.

This study has two key limitations. First, differences in volatiles emitted from traps were not measured. Visual and olfactory cues can interact to influence orientation behavior in necrophagous dipterans [6,7]. Hot weather during the study (mean, approximately 26 °C) may have caused differential decomposition rates and volatile release rates among traps of different colors [39,40]. We believe that it is unlikely that our results are entirely driven by color-dependent heating and bait volatilization though. If true, we would expect similar capture rates (consistently high or low) in the clear and yellow traps; however, these traps produced markedly different results. Future studies, however, should explicitly measure the temperature inside painted bottle traps of different colors to determine whether this is the case. In addition, paints might have also emitted volatiles that influenced attraction. To minimize these effects, all traps were painted at least five days before deployment to give the volatile organic compounds time to off-gas, and the same brand and finish of paint (Rustoleum satin) was used on all traps. Second, this study was conducted during a single sampling period. Future studies should replicate this experiment across multiple periods and test additional paint colors, brands, and finishes to determine how robust attraction to different colors is in adult necrophagous dipterans.

## 5. Conclusions

In conclusion, this study revealed that attraction to yellow is not consistent across necrophagous Diptera and that yellow traps may be less effective for sampling some taxa. This study provides evidence that trap color can influence the sampling of *L. coeruleiviridis*, a forensically important species, with clear traps yielding the highest captures and yellow traps the lowest. Although colored paper or sticky traps are commonly used to sample necrophagous dipterans, these results suggest that clear bottle traps in which the bait is visible may be preferable in some contexts. Additional studies incorporating rarer taxa, behavioral assays, and neurophysiological approaches would further clarify the role of color cues in the sampling of adult necrophagous dipterans.

## Figures and Tables

**Table 1:** Summary statistics from negative binomial generalized linear models examining the effects of block and trap color on the abundance of each taxon.

	Block (9 * DF)		Trap Color (3 DF)	
Dipteran Taxon	Wald χ^2^	*p*	Wald χ^2^	*p*
All Diptera	15.229	0.085	16.184	0.001
Calliphoridae	13.967	0.125	13.386	0.004
*Lucilia coeruleiviridis*	12.478	0.188	11.396	0.010
*Phormia regina*	7.153	0.413	3.084	0.379
*Cochliomyia macellaria*	1.416	0.493	3.455	0.063
Muscidae	12.036	0.211	17.088	<0.001
Sarcophagidae	8.592	0.198	10.893	0.012

* 7 DF for *P. regina,* 2 DF for *C. macellaria*, which were not collected in every block of traps.

## Data Availability

Dataset available on request from the authors.
